# Dietary Nitrate Supplementation Is Not Helpful for Endurance Performance at Simulated Altitude Even When Combined With Intermittent Normobaric Hypoxic Training

**DOI:** 10.3389/fphys.2022.839996

**Published:** 2022-03-14

**Authors:** Ana Sousa, João L. Viana, Jaime Milheiro, Vítor M. Reis, Grégoire P. Millet

**Affiliations:** ^1^Research Center in Sports Sciences, Health Sciences and Human Development (CIDESD), University of Trás-Os-Montes and Alto Douro, Vila Real, Portugal; ^2^Research Center in Sports Sciences, Health Sciences and Human Development (CIDESD), Instituto Universitário da Maia (ISMAI), Maia, Portugal; ^3^CMEP - Exercise Medical Center & Spa, Porto, Portugal; ^4^Olympic Committee of Portugal, Lisbon, Portugal; ^5^ISSUL, Institute of Sport Sciences and Physical Education (ISSEP), University of Lausanne, Lausanne, Switzerland

**Keywords:** nitrate, hypoxia, performance, intermittent training, endurance

## Abstract

**Introduction:**

Training intensity and nutrition may influence adaptations to training performed in hypoxia and consequently performance outcomes at altitude. This study investigates if performance at simulated altitude is improved to a larger extent when high-intensity interval training is performed in normobaric hypoxia and if this is potentiated when combined with chronic dietary nitrate (NO_3_^−^) supplementation.

**Methods:**

Thirty endurance-trained male participants were allocated to one of three groups: hypoxia (13% F_i_O_2_) + NO_3_^−^; hypoxia + placebo; and normoxia (20.9% F_i_O_2_) + placebo. All performed 12 cycling sessions (eight sessions of 2*6 × 1 min at severe intensity with 1 min recovery and four sessions of 4*6*10 s all-out with 20 s recovery) during a 4-week period (three sessions/week) with supplementation administered 3–2.5 h before each session. An incremental exhaustion test, a severe intensity exercise bout to exhaustion (*T*_lim_) and a 3 min all-out test (3AOT) in hypoxia (F_i_O_2_ = 13%) with pulmonary oxygen uptake ( $\dot{V}$ O_2_), $\dot{V}$ O_2_ kinetics, and changes in *vastus lateralis* local O_2_ saturation (SmO_2_) measured were completed by each participant before and after training.

**Results:**

In all tests, performance improved to the same extent in hypoxia and normoxia, except for SmO_2_ after *T*_lim_ (*p* = 0.04, *d* = 0.82) and 3AOT (*p* = 0.03, *d* = 1.43) which were lower in the two hypoxic groups compared with the normoxic one. Dietary NO_3_^−^ supplementation did not bring any additional benefits.

**Conclusion:**

Performance at simulated altitude was not improved to a larger extent when high-intensity interval training was undertaken in normobaric hypoxic conditions, when compared with normoxic training. Additionally, dietary NO_3_^−^ supplementation was ineffective in further enhancing endurance performance at simulated altitude.

## Introduction

Although most sport events are held at sea level, athletes may be required to compete in hypoxic environments (Burtscher et al., 2018); e.g., some stages of the road cycling “Tour de France,” mountain ultramarathon as the Hard Rock or Ultra Trail du Mont Blanc, ski mountaineering competitions, or soccer championship in Bolivia. Yet, altitude has a detrimental effect on aerobic performance (for a review see Millet et al., 2010). When acutely exposed to lower inspired oxygen (O_2_) pressure (inducing a lower O_2_ availability), a significant reduction of maximal oxygen uptake ( $\dot{V}$ O_2_max) occurs (Lawler et al., 1988). In contrast, anaerobic capacity is well maintained in hypoxia (Friedmann et al., 2007). When logistically impossible to live and/or train in real altitude conditions, intermittent training in normobaric/hypobaric hypoxia (i.e., living low training high, LLTH) may represent a reliable alternative to pre-acclimatize and prepare for competitions in altitude (Chapman et al., 2013). By adding the stress of hypoxia during both aerobic or anaerobic interval training, performance at sea level in endurance athletes may not increase any more than simply perform the same training at sea level (Faiss et al., 2013a). Benefits on sea-level performance, if training stimulus would be increased (allowing higher recruitment of fast twitch fibers), are still ambiguous (Lundby et al., 2012), and presently unknown when preparing athletes for altitude performance.

When athletes perform hypoxic training, several key components of the training program need to be adjusted; i.e., planning and periodization; training intensity; recovery; fatigue monitoring and acclimatization; hydration; and nutrition (Mujika et al., 2019). One nutrient is of particular interest: inorganic nitrate (NO_3_^−^) and a precursor of nitric oxide (NO), which is traditionally ingested by athletes in the form of beetroot juice. Studies conducted with moderately trained participants in normoxia conditions indicate that dietary NO_3_^−^ can improve muscle efficiency (by reducing the O_2_ cost of submaximal exercise) and enhance skeletal muscle contractile function. However, others have not been able to confirm these improvements with highly trained individuals (for a review see Jones et al., 2018). The dietary practice involving the serial reduction of NO_3_^−^ to nitrite (NO_2_^−^) and further to NO, a recently described pathway to generate NO (Lundberg et al., 1994), has gained popularity among athletes. Such interest results in the enhanced reduction of NO_2_^−^ to NO in conditions of acidosis (Modin et al., 2001) and hypoxia (Castello et al., 2006), suggesting that dietary NO_3_^−^ has the potential to improve training adaptation in hypoxic conditions.

Vanhatalo et al. (2011) showed that 0.75 L (9.3 mmol NO_3_^−^) of beetroot juice ingested during 24 h prior to hypoxic exposure (~14.5% O_2_—3,000 m) augmented time limit for single-leg knee extension high-intensity exercise, accompanied by a reduction in muscle metabolic perturbation in healthy subjects. Similarly, it was reported that 3 (8.4 mmol/day; Kelly et al., 2014) and 6 days (5.1 mmol/day; Masschelein et al., 2012) of beetroot juice induced longer time limit at 75%∆ cycling exercise (~13.1% O_2_–3,600 m) and in an incremental cycle test (~11.0% O_2_—5,200 m). The latter was also accompanied by a minor decrease in arterial saturation (SaO_2_). In contrast, trained cyclists have shown no beneficial effects on 15 km cycle time trial at ~11.0% O_2_ (5,200 m) after 3 days of sodium nitrate supplementation (0.1 mmol/kg/day; Bourdillon et al., 2015).

Given the lack of consistency in performance and metabolic improvements, dietary NO_3_^−^ supplementation could be interpreted as unnecessary to improve performance or training adaptation in hypoxic conditions. However, several factors may potentially contribute to a misinterpretation of this recommendation. It was previously suggested that extending the pre-competition supplementation period (at least up to 4 weeks) may benefit exercise performance (Wylie et al., 2016). Therefore, the short duration of NO_3_^−^ supplementation traditionally used in previous studies (~3–6 days) may not be sufficient to elicit the full ergogenic effect of NO_3_^−^. Moreover, the hypoxia-induced lowering of the training stimulus (especially for high-intensity exercise that requires greater input of type II fibers), that is, paramount for the NO_3_^−^—NO_2_^−^—NO bioavailability backup system enhancement (Castello et al., 2006), may have also influenced the effects on hypoxic training adaptations and consequently, performance outcomes in hypoxic environments. However, the effects of the combination of chronic dietary NO_3_^−^ supplementation and of high-intensity (i.e., in severe intensity domain and with repeated sprints) training in hypoxia on subsequent aerobic and anaerobic performances at altitude remain to be clearly investigated. Therefore, the aim of the present study was to investigate the influence of an extended dietary NO_3_^−^ supplementation period (4 weeks) through beetroot juice ingestion, combined with intermittent high-intensity exercise training sessions in hypoxia (3 days/week at ~3,000 m) on the performance at simulated altitude (~3,000 m). It was hypothesized that (i) performance at simulated altitude would be improved to a larger extent when intermittent high-intensity exercise training sessions are performed in hypoxia, when compared to similar training in normoxia and (ii) that chronic dietary NO_3_^−^ supplementation would potentiate a performance improvement, when compared to the placebo condition.

## Materials and Methods

### Participants

Thirty endurance-trained male participants, regularly involved in competitive running, cycling, and/or triathlon events (4–5 times per week: ~10–15 h) in the past 5 years, and volunteered to take part in this study. Exclusion criteria were as: (i) smoking or other chronic issues; (ii) using medication or dietary supplements in the past 3 months, or during the duration of the study; and (iii) being exposed to altitude (≥2,000 m) in the past 3 months. The Ethics Committee of the University of Trás-os-Montes and Alto Douro approved this study (Reference 14A/CE/2017) and all participants provided written informed consent after being informed of all experimental procedures and possible risks associated with the experiments. All procedures complied with the ethics code of the Declaration of Helsinki.

### Study Design

This randomized, single-blind, placebo-controlled, and independent group study was carried out in a laboratory at sea level (Porto’s Exercise Medical Center, Portugal), which allows controlling for humidity, temperature, altitude, and % O_2_ availability. This chamber reduced the fraction of O_2_ in the inspired air by diluting it with extra nitrogen (normobaric hypoxic system: b-Cat®). The oxygenation level in the chamber was controlled by a specific O_2_ sensor placed inside the chamber and remained stable in each training session/test. All participants had previously been involved with research in our laboratory and were familiar with the equipment and testing protocols implemented, thus familiarization sessions were not required. Consequently, $\dot{V}$ O_2_max values were already known, and participants were randomly assigned to one of three experimental groups (HNO, HPL, and CON) with similar distributions for $\dot{V}$ O_2_max, age, body mass, and height (55.1 ± 5.4, 57.3 ± 8.3, and 51.9 ± 7.9 ml·kg^−1^·min^−1^; 36.1 ± 4.6, 37.4 ± 5.7, and 34.9 ± 8.4 years old; 71.0 ± 7.7, 70.2 ± 7.3, and 73.2 ± 9.5 kg; and 175.7 ± 7.1, 173.3 ± 6.8, and 175.3 ± 6.9 cm, for HNO, HPL, and CON groups, respectively). Participants performed 12 high-intensity interval-training (HIIT) sessions during a 4-week period (three sessions/week, 48 h in-between each session), in one of three experimental groups: (i) HNO: high-intensity exercise training sessions in normobaric hypoxia (F_i_O_2_ = ~13%, ~3,000 m) with NO_3_^−^ supplement; (ii) HPL: high-intensity exercise training sessions in normobaric hypoxia (F_i_O_2_ = ~13%, ~3,000 m) with placebo supplement, and (iii) CON: high-intensity exercise training sessions in normoxia (F_i_O_2_ = 20.9%) with placebo supplement.

Participants were instructed to maintain their normal daily activity throughout the 4-week period, and only an extra training session peer week was allowed (except for week 4). Also, they were advised to maintain their normal diet although they received a list of foods rich in NO_3_^−^ to be avoided (e.g., leafy green vegetables, beetroot, and processed meats) to isolate supplemented NO_3_^−^ as a cause of any potential beneficial effect. Moreover, participants were asked to abstain from the use of any chewing gum and antibacterial mouthwash products, as these have been shown to inhibit the conversion of NO_3_^−^ to NO_2_^−^ by bacteria in the oral cavity. A food diary was used to ensure adherence to the intervention.

In the week before (pre-intervention) and after (post-intervention) the 4-week exposure, participants performed an incremental, continuous test to exhaustion (INC), an exercise transition from rest to severe intensity to exhaustion (*T*_lim_), and a 3 min all-out test (3AOT), in a cycle ergometer (Lode Excalibur Sport, Groningen, Netherlands) in normobaric hypoxia (F_i_O_2_ = ~13%, ~3,000 m).

### Training Intervention

From the 12 HIIT sessions, in each week, two sessions of short aerobic intervals (HIT; first- and third-week’s sessions) and one session of repeated-sprint training (RST; second week’s session) were performed on the cycle ergometer. A warm-up of 10 min at 100 W + 4 min at 50% of power associated with $\dot{V}$ O_2_max (p $\dot{V}$ O_2_max) and a cool-down period of 15 min of active recovery at 100 W were completed in each session. The main sets for HIT were as follows: two sets of 6 × 1 min at 90%∆ (90% between $\dot{V}$ O_2_max and the first ventilatory threshold), with 1 min active recovery between repetitions and 3 min between sets, both at 50% of p $\dot{V}$ O_2_max (short intervals). The main sets were for RST: four sets of 6 × 10 s all-out, with 20 s active recovery and 3 min between sets, both at 50% of p $\dot{V}$ O_2_max (repeated-sprint training). The number of repetitions was increased from 6 (first and second weeks) to 7 (third and fourth weeks) in both sessions. The training intensities used were relative to specific p $\dot{V}$ O_2_max (assessed in hypoxia for HNO and HPL, and in normoxia for CON group). For the total duration of all training sessions (ranging from 52 to 57 min), participants always remained inside the chamber. For the oxygenation level in the environmental chamber to remain stable, no one was allowed in or out of the chamber during the training sessions.

Participants were instructed to keep their cadence between 70 and 90 rpm in HIIT’s short intervals, but in the RST sessions, the cadence was not fixed (isokinetic operation mode of the ergometer) and therefore, encouragement was given to perform at their maximal effort. In addition to the three supervised high-intensity training sessions, participants could perform one extra training session (that occurred on weekend days, in every case) below the moderate intensity domain (~60 min) in weeks 1–3 at normoxic conditions. No extra training session was allowed in week 4 to eliminate acute physiological effects.

### Supplementation

Supplements were ingested 2.5–3 h prior to each session. NO_3_^−^ was administered in the form of beetroot juice, containing 400 mg of a powdered standardized beetroot extract (containing 2% of NO_3_^−^, ~8.4 mmol, according to the manufacturer label) dissolved in 150 ml of water (Sabeet®, Sabinsa Corporation). An equivalent volume of black currant juice, with negligible NO_3_^−^ content, was served as a control drink. Both supplements were similar in appearance and taste. Supplementation was extended until post-intervention period (ingested 2.5–3 h prior each performance test).

### Performance Tests

In INC test, which was always conducted first, both $\dot{V}$ O_2_max and p $\dot{V}$ O_2_max were assessed. This test consisted of a 2-min step duration with 30 W increments between steps with a self-selected cadence between 70 and 90 rpm. Time sustained was determined through the *T*_lim_ test and performed at 80%∆ (i.e., first ventilatory threshold—VT_1_, plus 80% of the difference between VT_1_ and $\dot{V}$ O_2_max). The test ended when the cadence could no longer be maintained within 10 rpm of the preferred cadence for >5 s. Critical power (CP) and the curvature constant (W′) were both assessed during the 3AOT test as previously described (Sousa et al., 2021). In this test, during the last 5 s of the baseline period, subjects were asked to increase their cadence to approximately 110 rpm. The braking resistance for the pedals to move was set (using the linear mode of the ergometer) so that the subjects would attain the power output halfway between $\dot{V}$ O_2_max and the VT_1_ (i.e., VT_1_ + 50%∆) on reaching their preferred cadence (linear factor = power/cadence squared).

In both *T*_lim_ and 3AOT tests (performed randomly), a standard 5 min warm-up exercise at 50% of p $\dot{V}$ O_2_max, followed by a 5-min passive rest, was performed. In all three tests, a standard 3-min period of unloaded baseline pedaling (8 W) at each participant’s preferred cadence, preceded each test. Verbal encouragement was given to motivate the participants to perform their best effort in the INC and 3AOT tests protocols and for as long as possible during the *T*_lim_ one. All tests were performed at room temperature (18–20°C) and 40–50% relative humidity (standardized and at the same time of the day (±2 h) for each participant to minimize an impact of the circadian variation on performance).

### Experimental Measures

During each test, respiratory and pulmonary gas-exchange variables were continuously measured in a breath-by-breath mode (K5, Cosmed, Italy). The gas analyzer was calibrated before each test with gases of known concentration (16% O_2_ and 5% CO_2_) and the turbine volume transducer calibrated with a 3-L syringe, according to the manufacturer’s instructions. $\dot{V}$ O_2_max (measured as the average of breaths over the last 30 s) was defined in one of the following cases: a plateau in $\dot{V}$ O_2_ despite an increase in power, [La-] ≥ 8 mmol·L^−1^, respiratory exchange ratio ≥1.0, HR >90% of [220—age], or a volitional exhaustion (controlled visually and case-by-case). p $\dot{V}$ O_2_max was estimated as the power corresponding to the first stage of the INC test that elicited $\dot{V}$ O_2_max. If a plateau of less than 2.1 ml·min^−1^·kg^−1^ could not be observed, the p $\dot{V}$ O_2_max was calculated as previously described (Kuipers et al., 1985). VT_1_ and VT_2_ were determined as previously suggested (Sousa et al., 2021). The percentage of power, $\dot{V}$ O_2_ and HR relative to p $\dot{V}$ O_2_max, $\dot{V}$ O_2_max, and HRmax associated to both VT_1_ and VT_2_ (VT_1_-W, VT_1_-O_2_, VT_1_-HR, VT_2_-W, VT_2_-O_2_, and VT_2_-HR) were considered for analysis.

During both *T*_lim_ and 3AOT tests, changes in local O_2_ saturation (SmO_2_), which represents the balance between O_2_ delivery and extraction by the muscle, and in total hemoglobin (THb), an indicator of local blood volume, were assessed in the capillaries through a near-infrared spectroscopy (NIRS) monitor (Moxy monitor, Fortiori Design, Minnesota, United States). The Moxy monitor was positioned on the participant’s dominant leg *vastus lateralis* muscle, halfway between the greater trochanter and lateral epicondyle of the femur, perpendicular to the muscle fiber orientation. This muscle was chosen as it is part of the knee extensor group which is the primary contributor to force generation in the crank of the bicycle during down stroke of the pedal. Prior to placement, this area was trimmed with an electric razor and cleaned with alcohol swabs. Moxy was secured with a light shield and black athletic tape to block ambient near-infra red light from interfering with the detectors. Its exact position was recorded and replicated in all tests (pre-and post-intervention periods). As suggested, skinfold thickness at the site of the Moxy placement was measured using a skinfold caliper (Harpenden Ltd.) to ensure that the skinfold thickness was <1/2 the distance between the emitter and the detector (25 mm).

Capillary blood samples (5 μl) were collected from the earlobe for whole lactate concentration ([La^−^]) determination before exercise (at rest) and immediately at the end of each test, during the 1st, 3rd, 5th, and 7th minute of the recovery period until maximal ([La^−^]_max_: for INC test) and peak values ([La^−^]_peak_: *T*_lim_ and 3AOT tests) were reached (Lactate Pro2, Arkay, Inc., Kyoto, Japan).

### Data Analysis

As the gas analyzer did not measure directly F_i_O_2_, the corrected F_i_O_2_ (~13%) was inputted at the end of each test. $\dot{V}$ O_2_ errant breaths (e.g., caused by swallowing, coughing, and signal interruptions) were omitted from the $\dot{V}$ O_2_ analysis by including only those that were between $\dot{V}$ O_2_ mean ± 4 SDs. After this process, the breath-by-breath data were used for $\dot{V}$ O_2_ kinetic analysis in the *T*_lim_ test. The first 20 s of data after the onset of exercise (cardio-dynamic phase) was not considered for model analysis. To allow comparison of the $\dot{V}$ O_2_ response, data were modeled using both a mono (Equation 1) and a double exponential approach (Equation 2) to isolate a possible $\dot{V}$ O_2_ fast component response.


(1) $${\mathrm{VO}}_{2}\;\left(t\right)=A_{0}+A_{1}*\left(1-\exp{\;}^{(-t/\tau_{1}}{}^{)}\right)$$

(2) $${\mathrm{VO}}_{2}\;\left(t\right)=A_{0}+A_{1}*\left(1-\exp{\;}^{(-t/\tau_{2}}{}^{)}\right)+A_{2}*\left(1-\exp{\;}^{(-t/\tau_{2}}{}^{)}\right),$$

where VO_2_(*t*) represents the relative VO_2_ at the time *t*, *A*_0_ is the VO_2_at rest (ml·kg^−1^·min^−1^), and *A*_1_ and *A*_2_ (ml·kg^−1^·min^−1^) and *τ*_1_ and *τ*_2_ (s) are the amplitudes and time constants of the fast and slow VO_2_ components, respectively. *A*_1_ was used to determine the gain (*G* = *A*_1_/power) of the primary component. To estimate VO_2_ kinetics parameters, equations were fitted to the exercise data (GraphPad Prism, GraphPad Software, La Jolla, CA, United States) by minimizing the sum of the mean squares of the differences between modeled and measured VO_2_ values.

Initially, the raw SmO_2_ and [THb] data were treated using a smooth spline filter to reduce the noise created by movement and data presented every 2 s. Baseline SmO_2_ (SmO_2base_) and baseline [THb] were computed as a 30 s average while subjects performed the standard 3 min period of unloaded baseline pedaling (8 W) at their preferred cadence before the beginning of each test. Minimum SmO_2_ (SmO_2min_) was the lowest 6 s average reached during each test. Maximum SmO_2_ (SmO_2max_) and maximum [THb] were the highest 6 s average reached during each test with recovery phase included. Average SmO_2_ from 30 to 120 s after the end of each test was used to assess recovery SmO_2_ (SmO_2recovery_). For each test, baseline SmO_2base_ and SmO_2min_ are expressed as % of SmO_2max_ (relative-SmO_2base_ and relative-SmO_2min_, respectively). Change in SmO_2_ (ΔSmO_2_) and change in [THb] ([ΔTHb]) were calculated as the difference between relative-SmO_2min_ and relative-SmO_2base_ and, the difference between maximal and baseline [THb].

### Statistical Analysis

A total of 10 participants per group were considered for a type I error of 5%, a power of 80%, with statistical significance, and an average to be detected with probability population effect size of 0.5 (as it indicates a moderate to large difference; G*Power software version 3.1.9.2). *A priori* type of power analysis was used considering the student’s *t*-test as the statistical test performed. Shapiro–Wilk test confirmed the data normality and Levene’s test the variance homogeneity. Data are presented as mean ± SD. A two-way (group × time) ANOVA test was used to test main and interaction effects for the studied variables in performance (*post-hoc* comparisons when appropriate: Bonferroni test). Student’s *t*-tests were used to test: (i) performance differences at moderate altitude when high-intensity exercise training sessions were performed at moderate altitude, compared to the condition where they were performed at normoxia (HNO + HPL vs. CON) and (ii) performance differences at moderate altitude when chronic dietary NO_3_^−^ supplementation was used together with high-intensity exercise training sessions performed at hypoxia, compared to the condition where placebo condition was used (HNO vs. HPL). Repeated measures *T*-Tests were used to test both pre- vs. post-performance differences in-between each intervention group. Pearson’s correlation coefficient (*r*) was employed to analyze the bivariate correlations between the different variables. Magnitude of the changes using effect sizes (ES) = (post-test mean − pretest mean)/(pretest standard deviation) was obtained for each test, and threshold values considered were “trivial” (<0.2), “small” (0.2–0.6), “moderate” (0.6–1.2), “large” (1.2–2.0), and “very large” (>2.0). Magnitudes of standardized effects (Cohen’s *d* calculated as the difference in means divided by the pooled SD) were determined against the following criteria: small, 0.2–0.5; moderate, 0.5–0.8, and large, >0.8. All statistical procedures were conducted with SPSS 24.0 and the significance level was set at 5%.

## Results

### Incremental Continuous Protocol Until Exhaustion Test

**Figure 1:**
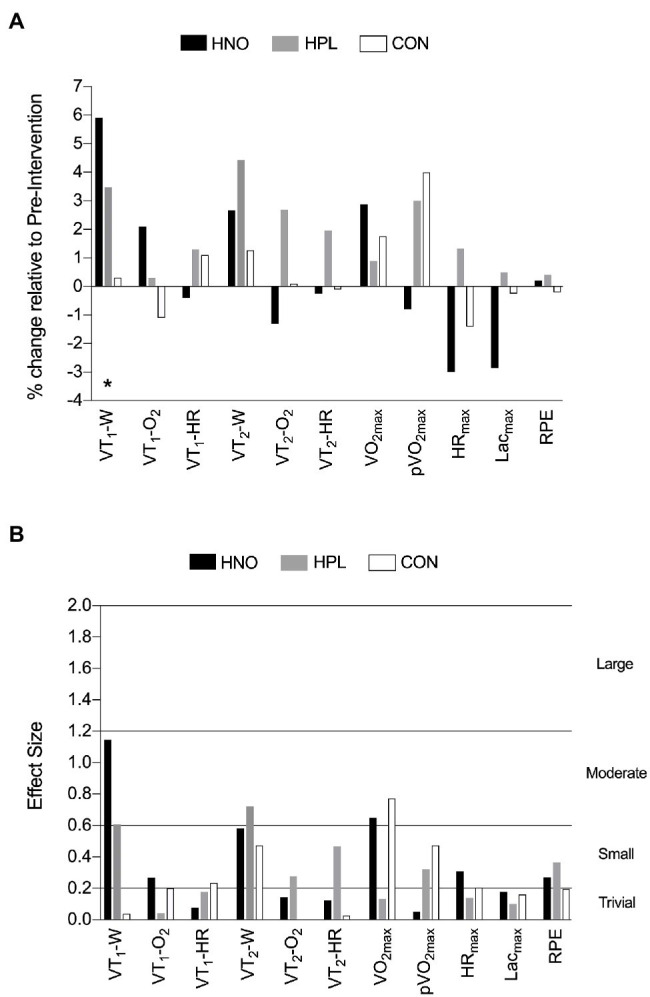
The effects of 4-weeks training in the INC test-related parameters presented as mean percentage of change relative to pre-intervention period (post-intervention mean—pre-intervention mean/pre-intervention mean*100): **(A)** and the corresponding mean effect size (post-intervention mean—pre-intervention mean/square root of the sum of the two standard deviations minus 2*correlation coefficient): **(B)** following high-intensity exercise training sessions performed in hypoxia with NO_3_^−^ (HNO: black), in hypoxia with placebo (HPL: gray), and in normoxia with placebo (CON: white). Threshold values considered for effect size were “trivial” (<0.2), “small” (0.2–0.6), “moderate” (0.6–1.2), and “large” (1.2–2.0). ^*^Differences between hypoxia (HNO + HPL) vs. normoxia (CON; *p* < 0.05).

Figure 1 shows the effects of 4 weeks of training in the INC test’ related parameters for all experimental groups. Except for VT_1_-W (time effect: *p* = 0.04) which was higher in hypoxia compared to normoxia (*p* = 0.03, *d* = 0.62), no other differences were found, inclusively between chronic dietary NO_3_^−^ supplementation vs. placebo condition. From all the INC test-related parameters, only VT_1_-W, $\dot{V}$ O_2_max (ES: 1.14 and 0.65, respectively, in HNO group), VT_2_-W (ES: 0.72 in HPL group), and $\dot{V}$ O_2_max (0.77 in CON group) percentage differences had a moderate effect, with the remaining one being small (0.2–0.6) and trivial (<0.2). From pre- to post-intervention period, VT_1_-W was significantly higher (*p* = 0.006, *d* = 0.48) and [Lac]_max_ significantly lower (*p* = 0.009, *d* = 0.46) in HNO group, and $\dot{V}$ O_2_max significantly higher (*p* = 0.037, *d* = 0.94, *r* = 0.7) in CON group. No significant differences were found for HPL group.

### Exercise Transition From Rest to Severe Intensity Until Exhaustion and 3 Min All-Out Tests

**Figure 2:**
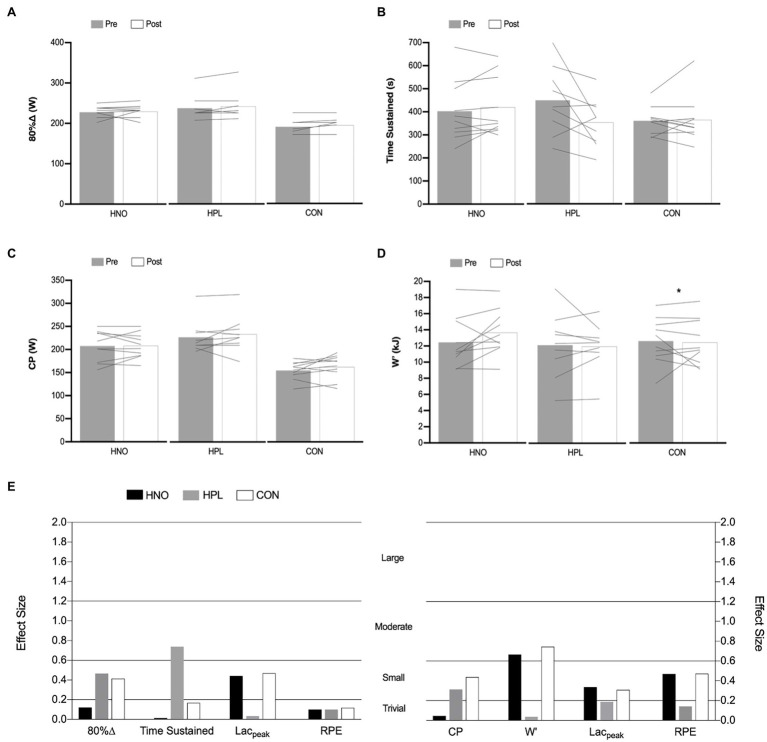
The effects of 4-weeks training in the 80%∆ intensity and time sustained of the *T*_lim_ test **(A,B)** and on the CP and W′ of the 3AOT **(C,D)** presented as mean (columns) and individual values (lines) in both pre- (gray) and post-intervention (white) periods following high-intensity exercise training sessions performed in hypoxia with NO_3_^−^ (HNO), in hypoxia with placebo (HPL) and in normoxia with placebo (CON). Mean corresponding effect size (post-intervention mean—pre-intervention mean/square root of the sum of the two standard deviations minus 2*correlation coefficient) for HNO (black), HPL (gray), and CON (white) groups in both *T*_lim_ and 3AOT-related parameters are presented in **(E)**. Threshold values considered for effect size were “trivial” (<0.2), “small” (0.2–0.6), “moderate” (0.6–1.2), and “large” (1.2–2.0). ^*^Differences between pre- and post-intervention period (*p* < 0.05).

Figure 2 shows the effects of 4 weeks of training in the *T*_lim_ and 3AOT-related parameters for all experimental groups. No differences were found between hypoxia vs. normoxia intervention and between chronic dietary NO_3_^−^ supplementation vs. placebo condition in any of the tests. Only time sustained (ES: 0.74, in HPL) and W′ (ES: 0.67 and 0.74 in HNO and CON groups, respectively) differences had a moderate effect, with the remaining one being small (0.2–0.6) and trivial (<0.2). From pre- to post-intervention period, W′ was significantly lower (*p* = 0.04, *d* = 0.74, *r* = 0.86) in CON group, and no differences were found for both HNO and HPL groups.

Table 1 shows the mean values for the $\dot{V}$ O_2_ kinetics’ parameters obtained during the *T*_lim_ test. In the pre-intervention period, $\dot{V}$ O_2_ kinetics’ analysis showed that from the 30 participants, only nine individual responses (30%) were better modeled using a double exponential approach (*A*_2_ = 13.03 ± 3.4 ml·kg^−1^·min^−1^: mean ± SD) with an overall *R*^2^ = 0.85 ± 0.06. In the post-intervention period, only eight individual responses (~27%) were better modeled using a double exponential approach (*A*_2_ = 12.66 ± 4.1 ml.kg^−1^.min^−1^: mean ± SD) with an overall *R*^2^ = 0.85 ± 0.09. For comparison purposes, only common $\dot{V}$ O_2_ kinetics’ parameters were presented in Table 1.

**Table 1:** Mean ± SD values for the $\dot{V}$ O_2_ kinetics’ parameters obtained in pre- and post-intervention periods during *T*_lim_ for high-intensity exercise training sessions performed in hypoxia with NO_3_^−^ (HNO), in hypoxia with placebo (HPL), and in normoxia with placebo (CON).

Parameters	Intervention	Groups		
		HNO	HPL	CON
*A*_0_ (ml·kg^−1^·min^−1^)	Pre	16.1 ± 0.9	15.4 ± 1.0	17.2 ± 1.0
	Post	16.4 ± 1.3	15.1 ± 0.9	17.0 ± 0.9
*A*_1_ (ml·kg^−1^·min^−1^)	Pre	25.0 ± 1.3	24.8 ± 1.0	23.4 ± 1.3
	Post	24.6 ± 1.9	25.8 ± 2.2	23.4 ± 1.7
*τ*_1_ (s)	Pre	39.6 ± 1.7	39.1 ± 1.6	42.1 ± 1.9
	Post	39.6 ± 1.9	39.6 ± 1.8	42.5 ± 1.4
Gain (ml·kg^−1^·min^−1^/W)	Pre	11.1 ± 0.6	10.5 ± 0.6	12.3 ± 0.8
	Post	10.9 ± 1.0	10.8 ± 0.9	12.1 ± 0.9

*A*_0_ is the VO_2_ at rest (ml·kg^−1^·min^−1^), *A*_1_ (ml·kg^−1^·min^−1^), and *τ*_1_ (s) are the amplitudes and time constants of the fast VO_2_ components, respectively. *A*_1_ was used to determine the gain (*G* = *A*_1_/power) of the primary component.

No differences were found between hypoxia vs. normoxia intervention and between chronic dietary NO_3_^−^ supplementation vs. placebo condition. $\dot{V}$ O_2_ kinetics’ parameters differences were all trivial (ES < 0.2) for every experimental group. From pre- to post-intervention period, no differences were found for any experimental group.

Table 2 shows the mean values for the oxygen saturation’ parameters obtained during the *T*_lim_ test in both pre- and post-intervention periods. Except for SmO_2recovery_ (group main effect: *p* = 0.001, *d* = 0.4), which was significantly higher in hypoxia compared with normoxia (*p* = 0.04, *d* = 0.82), no other differences were found, inclusively between chronic dietary NO_3_^−^ supplementation vs. placebo condition. The 4 weeks of training induced an increase in both SmO_2min_ (*p* = 0.04) and Relative-SmO_2min_ (*p* = 0.05) in HNO group (ES = 0.72 and 0.67, respectively). Moreover, CON group presented significantly higher SmO_2base_ mean values in the post-intervention period (*p* = 0.03, ES = 1.05). No differences were found for the HPL group.

**Table 2:** Mean ± SD values for the oxygen saturation’ parameters obtained during the *T*_lim_ for high-intensity exercise training sessions performed in hypoxia with NO_3_^−^ (HNO), in hypoxia with placebo (HPL), and in normoxia with placebo (CON).

Parameters	Intervention	Groups		
		HNO	HPL	CON
SmO_2base_ (%)	Pre	59.2 ± 8.8	58.6 ± 12.3	50.1 ± 7.3^#^
	Post	58.8 ± 9.3	59.0 ± 11.7	54.9 ± 9.9
SmO_2min_ (%)	Pre	2.8 ± 2.3^#^	3.1 ± 1.9	3.5 ± 1.2
	Post	6.33 ± 4.8	2.1 ± 0.4	5.25 ± 2.6
SmO_2max_ (%)	Pre	71.2 ± 7.5	66.1 ± 10.9	64.1 ± 10.1
	Post	67.8 ± 13.4	64.0 ± 13.2	61.8 ± 6.9
SmO_2recovery_ (%)^*^	Pre	57.3 ± 13.6	51.1 ± 18.7	39.6 ± 14.7
	Post	56.6 ± 11.1	54.9 ± 13.4	27.1 ± 15.5
Relative-SmO_2base_ (% of SmO_2max_)	Pre	83.4 ± 11.7	88.2 ± 6.8	79.1 ± 11.5
	Post	87.8 ± 10.8	92.7 ± 5.6	88.7 ± 12.1
Relative-SmO_2min_ (% of SmO_2max_)	Pre	3.80 ± 1.7^#^	3.4 ± 1.6	3.7 ± 1.5
	Post	10.1 ± 8.9	6.2 ± 4.1	6.6 ± 3.9
ΔSmO_2_ (%)	Pre	−79.7 ± 13.05	−68.8 ± 35.2	−70.2 ± 11.7
	Post	−77.7 ± 12.6	−70.2 ± 36.2	−74.3 ± 12.7
[ΔTHb] (AU)	Pre	0.11 ± 0.01	0.14 ± 0.01	0.13 ± 0.09
	Post	0.11 ± 0.05	0.14 ± 0.03	0.10 ± 0.05

SmO_2base_, SmO_2min_, SmO_2max_, and SmO_2recovery_: Baseline, minimum, maximum, and recovery SmO_2base_, respectively; Relative-SmO_2base_ and Relative-SmO_2min_: SmO_2base_ and SmO_2min_ expressed as % of SmO_2max_, respectively; ΔSmO_2_ and [ΔTHb]: change in SmO_2_ and [THb], respectively.

* Differences between hypoxia (HNO + HPL) vs. normoxia (CON).

# Differences between pre- and post-intervention period (*p* < 0.05).

Table 3 shows the mean values for the oxygen saturation’ parameters obtained during the 3AOT in both pre- and post-intervention periods. Except for SmO_2recovery_ (group main effect: *p* = 0.008, *d* = 0.2; interaction effect: *p* = 0.02, *d* = 0.2) which was significantly higher in hypoxia compared with normoxia (*p* = 0.03, *d* = 1.43), no other differences were found, inclusively between chronic dietary NO_3_^−^ supplementation vs. placebo condition. In HPL group, the 4 weeks of training induced an increase in SmO_2base_ (*p* = 0.001, ES = 1.5), Relative-SmO_2base_ (*p* = 0.05, ES = 1.14), and ΔSmO_2_ (*p* = 0.013, ES = 1.32). Moreover, CON group presented significantly higher SmO_2base_ (*p* = 0.04, ES = 0.79) and Relative-SmO_2base_ (*p* = 0.016, ES = 1.11) mean values in the post-intervention period compared to baseline. No differences were found for the HNO group.

**Table 3:** Mean ± SD values for the muscle oxygenation parameters obtained during the 3AOT for high-intensity exercise training sessions performed in hypoxia with NO_3_^−^ (HNO), in hypoxia with placebo (HPL), and in normoxia with placebo (CON).

Parameters	Intervention	Groups		
		HNO	HPL	CON
SmO_2base_ (%)	Pre	61.9 ± 10.5	57.2 ± 10.8^#^	50.1 ± 8.9^#^
	Post	63.1 ± 6.5	61.7 ± 11.3	59.8 ± 11.2
SmO_2min_ (%)	Pre	2.7 ± 2.1	4.5 ± 3.2	4.9 ± 3.1
	Post	5.4 ± 2.3	3.1 ± 1.8	6.9 ± 3.5
SmO_2max_ (%)	Pre	69.1 ± 6.8	67.8 ± 9.4	59.3 ± 12.9
	Post	69.1 ± 11.8	63.8 ± 10.9	66.4 ± 7.5
SmO_2recovery_ (%)^*^	Pre	60.7 ± 8.9	56.7 ± 10.9	36.7 ± 15.6^#^
	Post	56.9 ± 16.3	54.1 ± 12.9	53.9 ± 6.7
Relative-SmO_2base_ (% of SmO_2max_)	Pre	89.4 ± 9.6	84.5 ± 11.8^#^	85.7 ± 12.0
	Post	92.6 ± 10.8	96.8 ± 8.3	92.1 ± 8.8
Relative-SmO_2min_ (% of SmO_2max_)	Pre	4.06 ± 3.2	6.5 ± 3.1	8.4 ± 3.4
	Post	8.11 ± 3.1	4.9 ± 2.4	10.2 ± 4.2
ΔSmO_2_ (%)	Pre	−85.4 ± 12.3	−65.1 ± 35.9^#^	−77.3 ± 17.6
	Post	−84.48 ± 14.3	−76.83 ± 41.1	−80.2 ± 11.8
[ΔTHb] (AU)	Pre	0.12 ± 0.1	0.19 ± 0.1	0.13 ± 0.1
	Post	0.09 ± 0.06	0.07 ± 0.05	0.1 ± 0.09

SmO_2base_, SmO_2min_, SmO_2max_, and SmO_2recovery_: Baseline, minimum, maximum, and recovery SmO_2base_, respectively; Relative-SmO_2base_ and Relative-SmO_2min_: SmO_2base_ and SmO_2min_ expressed as % of SmO_2max_, respectively; ΔSmO_2_ and [ΔTHb]: change in SmO_2_ and [THb], respectively.

* Differences between hypoxia (HNO + HPL) vs. normoxia (CON).

# Differences between pre- and post-intervention period (*p* < 0.05).

## Discussion

The present study is the first investigation to analyze the influence of an extended dietary NO_3_^−^ supplementation period combined with high-intensity training sessions at simulated altitude on the aerobic and anaerobic performance at simulated altitude. Contradicting our initial hypothesis, we did not observe that performance at simulated altitude is improved to a larger extent when training sessions are performed in hypoxia, compared to the same training in normoxia, as no differences in many of the variables studied were found. Moreover, our second hypothesis was also not confirmed as we did not find evidence that chronic dietary NO_3_^−^ supplementation potentiates performance at simulated altitude.

### Training in Hypoxia

Although some improvements in anaerobic performance have been reported (Daniels and Oldridge, 1970; Gore et al., 1998; Friedmann et al., 2005; studies “uncontrolled”), it is extensively reported that no additional benefit on the aerobic performance of endurance athletes occurs when conducting interval training in a hypoxic environment, compared with the same training performed in normoxia (see Faiss et al., 2013a for a review). To overcome these limitations, it was recently proposed a new hypoxic training method: RST in hypoxia (RSH; Faiss et al., 2013b). As the training stimulus is maximal, allowing one to maintain high fast twitch fibers recruitment, additional positive results on performance could be expected when hypoxic stimulus is added. The consensus is that RSH leads to superior (1–5%) repeated-sprint ability in normoxic conditions, while larger increase in $\dot{V}$ O_2_max was not observed (Brocherie et al., 2017).

In a protocol similar to ours (eight RSH sessions during a 4-week period: 3*10 s all-out with 20 s and 5 min recovery between repetition and sets, respectively), Faiss et al. (2013b) showed that RSH (~at 3,000 m) delayed fatigue during a repeated-sprint test to exhaustion. However, endurance performance (during a 3AOT) was not increased from pre- to post-intervention period in either group, neither hypoxic stimulus evidenced larger improvements compared to the same training performed in normoxia (for both 3AOT and Wingate test). Also, a 5-week intervention of RSH (30 s sprints interspersed by 4.5 min recovery intervals, 3 weekly sessions, 4–6 sprints per session), performed at ~2,750 m altitude, did not alter endurance ( $\dot{V}$ O_2_max test and 30-min simulated time trial) or sprinting (30 s sprint test) performance to a higher extent compared to the same training stimulus performed in normoxia (De Smet et al., 2016). Even when exposure time was extended to 7 weeks (two sessions of high-intensity at 100 or 90% of p $\dot{V}$ O_2_max), Roels et al. (2005) did not report a greater increase in performance (submaximal cycling test, 10 min cycle time trial and incremental test to exhaustion).

In line with most of the previous studies, we did not find differences in performance between hypoxic and control groups, except for VT_1_-W in INC test (*p* = 0.09, *d* = 0.62). This was significantly higher when subjects were exposed to interval training in hypoxia compared to normoxia, achieving a moderate effect (ES = 1.14). In fact, only HNO group evidenced significantly higher VT_1_-W (*d* = 0.48) and lower [Lac]_max_ mean values (*d* = 0.46) from pre- to post-intervention period. Moreover, in both *T*_lim_ and 3AOT, local O_2_ saturation in the recovery period (SmO_2recovery_) was significantly higher in hypoxia compared with the control group (*d* = 0.82 and 1.43, respectively). Therefore, the participants who were exposed to the hypoxic stimulus during the 4-weeks intervention were able to recover O_2_ saturation faster after *T*_lim_ and 3AOT ended. Of interest, the addition of a single RSH session per week did not bring additional benefits and collectively, the combination of high-intensity sessions in hypoxia used in the present study (two HIT sessions and one RSH session) did not bring an additional aerobic and anaerobic performance enhancement compared to the same program performed in normoxia. However, an innovative and unexpected output of the present study is that this combination was also non effective for preparing athletes for simulated altitude performance.

There were also no differences in $\dot{V}$ O_2_ kinetics’ parameters assessed in *T*_lim_. It was previously reported that *τ*_1_ and A_2_ are highly plastic and decrease substantially with endurance exercise training in previously untrained individuals and that these adaptations are remarkably rapid (7–14 days; Womack et al., 1995). The authors proposed that the initial speeding (faster $\dot{V}$ O_2_ kinetics) might be related to faster muscle blood flow kinetics or increased capillary-to-fiber ratio. In our study, at least for the HNO group, SmO_2min_ was significantly lower in *T*_lim_ at post-compared to pre-intervention period. Although acute exposure to simulated altitude induces a slower $\dot{V}$ O_2_ kinetics (Sousa et al., 2021), there are no studies that analyzed the chronic impact of hypoxic exposure on altitude’ $\dot{V}$ O_2_ kinetics performance.

The lack of performance differences found in our study may be due to several factors. It has been purported that intermittent exposure and/or training under hypoxic conditions may enhance exercise sea-level performance through increasing circulating erythropoietin levels and hemoglobin mass, upregulation of hypoxia-inducible factor 1α (causing structural changes within the muscle fiber), and ventilatory adaptation (Chapman et al., 2013). However, intermittent training in hypoxia is quite likely to have a minimal effect on erythropoiesis since a large “hypoxic dose” is required for significantly “stimulating the erythropoietic pathway” to the point that it enhances post-altitude sea-level endurance performance [Levine and Stray-Gundersen, 2006; e.g., ≥3,000 m for at least 3 h/day for 1–3 weeks (Katayama et al., 2003)]. Intermittent training in hypoxia has more potential to induce muscle skeletal and ventilatory adaptations (Faiss et al., 2013a), being suggested that an exposure altitude ≥4,000 m for at least 1.5 h/day repeated for 5–6 days is required to stimulate ventilatory acclimatization and concomitant improvements in performance (Muza, 2007). Moreover, greater buffering capacity, lactic acid tolerance, and/or O_2_ extraction in the working muscle can be expected as well (Brocherie et al., 2017).

Collectively, it seems that the simulated altitude used in our study (~3,000 m), as well as the exposure time (~3 h per week for 4 weeks), may have been insufficient to promote the total ergogenic effect of training in hypoxia, either through hematological changes (hemoglobin mass) or *via* ventilatory acclimatization. It is also possible that the high-intensity exercise stimulus, which necessarily induces a higher acidosis, has blunted the erythropoiesis, making HIIT less effective, as previously suggested (Millet et al., 2010).

### Training in Hypoxia Combined With Dietary NO_3_^−^ Supplementation

It is well documented that the one-electron reduction of NO_2_^−^ to NO is enhanced in conditions of hypoxia (Kelly et al., 2014). Therefore, it would be expected that lowering skeletal muscle oxygenation would augment the potential for dietary NO_3_^−^ supplementation to improve physiological responses during exercise and performance at simulated altitude. However, we did not find evidence that chronic dietary NO_3_^−^ supplementation potentiates simulated altitude performance’ responses when high-intensity training sessions were held at moderate altitude, as no differences were found between HNO and HPL groups in any of the variables studied. This latter corroborates previous reports where acute NO_3_^−^ supplementation has shown to be ineffective in enhancing cycling and running’ performance in hypoxia (Arnold et al., 2015; Bourdillon et al., 2015). However, significant results were also shown for single-leg knee extension high-intensity (Vanhatalo et al., 2011) and cycling exercise (Masschelein et al., 2012; Kelly et al., 2014). Some factors may potentially contribute to the lack of agreement in these data, namely, the supplementation period (Wylie et al., 2016), dosage used (Wylie et al., 2013), and training status of the participants (Wilkerson et al., 2012).

With a protocol of 6 weeks in duration (five training sessions/week, 5 × 30 min/week at 4–6 mmol/L blood lactate), Puype et al. (2015) concluded that dietary NO_3_^−^ supplementation did not enhance the effects of intermittent hypoxic training on endurance exercise performance at sea level. However, the authors pointed out that higher daily doses of NO_3_^−^ (in contrast with the 0.07 mmol NO_3_^−^/kg body weight used) and administered as a bolus 2–3 h (and not 2.5–2 h) prior to the training sessions could result in significant training adaptations. With a protocol similar to ours (5 weeks: 4–6*30-s sprints interspersed by 4.5 min recovery intervals; three sessions/week), and a NO_3_^−^dosage of 6.45 mmol administered 3 h before each session, De Smet et al. (2016) showed that performance at sea level increased in all experimental groups (with and without NO_3_^−^ supplementation) to a similar extent. The only exception was that NO_3_^−^ supplementation combined with training sessions performed in hypoxia increased the proportion of type IIa fibers in muscle. Taken together, it would appear that even when chronic NO_3_^−^ supplementation is combined with HIIT training sessions performed in hypoxia, no effect on performance at sea level in healthy and/or moderately trained participant is observed. Our results extend these findings by suggesting that dietary NO_3_^−^ does not have the potential to improve training adaptation even at simulated altitude conditions. Therefore, given the lack of consistent outcomes, at least for well-trained endurance athletes, we suggest that chronic dietary NO_3_^−^ supplementation cannot be currently recommended to improve performance or training adaptation in hypoxic conditions, even when combined with HIIT training sessions, as previously suggested (Mujika et al., 2019).

One important feature of the present study has to do with an O_2_ “sparing-effect” of oral NO_3_^−^ supplementation, which was initially demonstrated by Larsen et al. (2007). In addition to liberating bioactive NO, NO_2_^−^ acts as both a potent vasodilator in hypoxia and as an alternative electron acceptor, replacing O_2_ in respiration (Bourdillon et al., 2015). Therefore, it would be expected the O_2_ “sparing effect” of NO_3_^−^ supplementation to be reflected in muscle tissue oxygenation in hypoxic conditions. In fact, our results show that from pre- to post-intervention period only the HNO group increased SmO_2min_ (from ~3 to 6%), at least in *T*_lim_ test, with the remaining experimental groups not evidencing this difference in any of the tests performed. These findings were previously observed by Masschelein et al. (2012) during steady-state cycling where an improvement in muscle tissue oxygenation index (and reduced HHb) during hypoxic exercise after 6 days of oral NO_3_^−^ supplementation occurred. In this present study, as performance was not significantly different between HNO and HPL groups, we attribute the greater improvement in SmO_2min_ to a better matching of O_2_ delivering to the metabolic requirements of the working muscles in *T*_lim_ test.

### Limitations

In our assay, we did no measure total baseline hemoglobin mass (Hb_mass_) or both plasma [NO_2_^−^] and [NO_3_^−^]. Although baseline Hb_mass_ influence on the potential increase of total Hb_mass_ after exposure to a Living High-Training Low design is unclear and remains debated (Millet et al., 2019), this may have influenced the results of the present study. In recent years, several studies have assessed the acute and chronic effect of NO_3_^−^ supplementation and reported increases in plasma [NO_3_^−^] (~500%) and [NO_2_^−^] (~40–140%; see Jones et al., 2018 for more details). Participants were instructed to maintain their normal diet, but they chose their own meals and were responsible for keeping their diets consistent. These could have influenced the effects of NO_3_^−^ supplementation on the outcome variables. Moreover, the well-trained status of the participants recruited, and consequently, their higher plasma NO_2_^−^ and NO_3_^−^ concentrations, may have played an influential role (Wilkerson et al., 2012). In support of this, data from Bescos et al. (2012) showed that 7 out of 13 trained endurance athletes were non-responders, with only small elevations in plasma NO_3_^−^ concentration observed following a 3-day supplementation protocol. Moreover, well-trained athletes experience less severe localized hypoxia and acidosis in the muscle compared with untrained populations at normoxia conditions (Wilkerson et al., 2012). However, when exposed to hypoxic environments, decreasing pulse oxygen saturation (SpO_2_) is larger in endurance-trained athletes (compared to less trained counterparts) due to the prevalence of exercise-induced hypoxemia caused by a higher cardiac output (Mollard et al., 2007). Collectively, this seems to suggest that the training status of some of our participants may have influenced any NO_3_^−^ supplementation effects or at least, expressed them differently. The fact that only male participants were part of this study reduces the applicability of the results found since our results are relevant to 50% of the population at large. Considering these latter, an important future direction for research is to investigate the moderating effect of training status and gender on the response to NO_3_^−^ supplementation in hypoxic conditions. Moreover, the inclusion of another experimental group (normoxia + supplementation) could have helped in the interpretation of the results obtained. Other sources of inter-individual variability are likely to be influential. Consequently, the study design would have been improved by better controlling these factors, particularly for the allocation of the participants in the different groups.

## Conclusion and Implications

The current experiment did not provide evidence that performance at simulated altitude (~3,000 m) would be improved to a larger extent, with high-intensity training sessions held at ~3,000 m simulated altitude for 4 weeks, compared to the condition where the training design was held in normoxia. In line with most of the previous studies on interval training in hypoxia providing no additional benefits, when compared to interval training in normoxia, the present study also reported that the weekly addition of a single RSH session was not sufficient to promote differences in performance. Also, the present study failed to show that dietary supplementation in NO_3_^−^ would further improve performance in simulated altitude than with a placebo. As many well-trained endurance athletes spend some of their preparation time under hypoxic environments (normobaric or hypobaric hypoxia) to compete at moderate altitude conditions, this study will assist athletes, practitioners, and coaches to consider the best LLTH design (longer exposure periods) and nutritional protocols (NO_3_^−^ supplementation currently not recommended) to improve performance or training adaptation in hypoxic conditions.

## Data Availability Statement

The raw data supporting the conclusions of this article will be made available by the authors, without undue reservation.

## Ethics Statement

The studies involving human participants were reviewed and approved by Ethics Committee of the University of Trás-os-Montes and Alto Douro approved this study (Reference 14A/CE/2017). The patients/participants provided their written informed consent to participate in this study.

## Author Contributions

AS, VR, and JV: conception and design of the work. AS, JM, JV, and GM: acquisition, analysis, and interpretation of data for the work. AS, VR, JM, and GM: drafting of the work and revising it critically for important intellectual content. All authors approved the final version of the manuscript and agree to be accountable for all aspects of the work in ensuring that questions related to the accuracy or integrity of any part of the work are appropriately investigated and resolved. All persons designated as authors qualify for authorship, and all those who qualify for authorship are listed.

## Funding

This work received funding from the Portuguese Foundation for Science and Technology, I.P. (SFRH/BPD/114670/2) and under the project UID04045/2020. AS was supported by the Portuguese Foundation of Science and Technology (SFRH/BPD/114670/2) and Research Center in Sports Sciences, Health Sciences and Human Development (CIDESD), is supported by the Portuguese Foundation of Science and Technology (UID/04045/2020).

## Conflict of Interest

The authors declare that the research was conducted in the absence of any commercial or financial relationships that could be construed as a potential conflict of interest.

## Publisher’s Note

All claims expressed in this article are solely those of the authors and do not necessarily represent those of their affiliated organizations, or those of the publisher, the editors and the reviewers. Any product that may be evaluated in this article, or claim that may be made by its manufacturer, is not guaranteed or endorsed by the publisher.
